# Online and offline dating violence: same same, but different?

**DOI:** 10.1186/s41155-024-00293-3

**Published:** 2024-04-11

**Authors:** Joana Jaureguizar, Maria Dosil-Santamaria, Iratxe Redondo, Sebastian Wachs, Juan M. Machimbarrena

**Affiliations:** 1 Department of Developmental and Educational Psychology, Faculty of Education, University of the Basque Country (UPV/EHU), Leioa, Spain; 2 Educational Sciences, University of the Basque Country, Leioa, Spain; 3 Department of Education and Social Studies, University of Münster, Munster, Germany; 4 Department of Clinical and Health Psychology and Research Methodology, Faculty of Psychology, University of the Basque Country (UPV/EHU), Avda. Tolosa 70, Donostia-San Sebastián, Gipuzkoa 20018 Spain

**Keywords:** Dating violence, Online, Offline, Victimization, Perpetration, Roles

## Abstract

**Background:**

Violent behaviors in romantic relationships among adolescents and young people are pressing social matter as they have an effect on both victims and aggressors. Moreover, in the last decades, new forms of harassment, control, and abuse through social networks and mobile phones have arisen. Therefore, now forms of online and offline dating violence coexist.

**Objectives:**

The aim was to analyze the prevalence rates by sex and age and the co-occurrence of online and offline dating violence. Moreover, the roles of online and offline dating violence aggressors and victims for their self-esteem, hostility, general psychological state, and emotional intelligence were investigated.

**Method:**

Three hundred forty-one university students from the Basque Country, Spain, participated in the study. They completed six validated instruments related to the mentioned variables.

**Results:**

Results highlight the high prevalence of online and offline dating violence in the sample and the co-occurrence of both types. No gender nor sex differences were found for online and offline dating violence perpetration and victimization. The correlation between online and offline dating violence was confirmed, and the reciprocity of violence is greater for offline violence. In relation to the role, both types of victims (online and offline) showed higher levels of hostility and psychological symptomatology than non-victims, but differences in self-esteem and emotional regulation were found in these modalities. Online and offline perpetrators shared hostility and some psychological symptoms as characteristics compared to non-victims, but differed in other symptoms and emotional intelligence.

**Conclusion:**

There is a continuum between offline and online victimization perpetration albeit differences in the characteristics such as self-esteem, emotional intelligence, and general functioning exist.

## Introduction

Violent behaviors in romantic relationships among adolescents and young people have become a priority area for analysis and intervention (Cascardi & Avery-Leaf, 2015; Muñoz-Rivas et al. 2019; O'Leary & Slep, 2012; Sunday et al. 2011; Ybarra et al. 2016), as can be seen, for example, from the variety of actions and laws that have been passed in recent years, for example by the US Congress (Offenhauer & Buchalter, 2011), or the educational intervention projects developed in schools of secondary education in many European countries, such as Italy, Poland, Portugal, Romania, Spain, and the UK (Vives-Cases et al. 2019).

Teen dating violence might be considered a public health problem with significant social, physical, and psychological implications (Park & Kim, 2018). Intimate partner violence does not only occur in stable adult couples, but scientific evidence shows that it occurs from an early age, with the first couple relationships in adolescents, perpetuating some behaviors that continue into adulthood (Taquette & Monteiro, 2019). Dating violence can be relational abuse (acts to humiliate or isolate the victim), verbal-emotional (manipulation or aggression using language), psychological (manipulation to humiliate and undermine the self-esteem of the victim, in public or private), physical (use physical force for the purpose of inflicting pain or suffering), and sexual (every non-desired sexual act, including comments that are sexual in nature) (Ali et al. 2016). Research indicates that verbal-emotional violence is the most frequent and accepted subtype among young couples (Carrascosa et al. 2018; Cornelius & Resseguie, 2007; Herbet et al. 2019; Rey-Anacona, 2008; Pazos et al. 2014; Wolfe et al. 2004). Other studies point out that relational violence precedes verbal-emotional and physical violence (Magdol et al. 1997; Schwartz et al. 2004).

In addition to these forms of abuse in adolescent couples, in recent years, other forms of harassment, control, and abuse have been added, derived from new forms of interaction through mobile phones and social networks: online dating violence (Borrajo & Gámez-Guadix, 2016; Draucker & Martsolf, 2010; Fernet et al. 2019; Zweig et al. 2013). Regarding types of online dating violence, a distinction between direct aggression (threats, insults, and public humiliation through online comments or images) and control (control of partner using the mobile phone or social networks) should be made (Muñiz, 2017). Thus, previous studies with university students found that online controlling behaviors are more common (ranges between 41 and 82%) than direct online aggressions (ranges around 10–14%) (Borrajo et al. 2015; De Los Reyes et al. 2022).

Moreover, online violence co-occurs with experiences of offline violence, so it is important to investigate both scenarios (Leisring & Giumetti, 2014; Temple et al. 2016; Viejo et al. 2016) and elucidate whether offline and online dating violence are two sides of the same reality or whether they are two different types of intimate partner violence. To this end, and on the basis of personality and typology theories, that suggest that violence is explained by a variety of personal variables (Dardis et al. 2015), the aim of the present study is to provide information about individual-level differences in offline and online dating violence perpetration and victimization.

### Prevalence of offline and online violence by sex and age

As far as dating violence prevalence in boys and girls is concerned, the findings from the large body of research examining gender differences are mixed and inconclusive, due to the different methodologies used, sample characteristics (women generally participate more in this type of study) and social desirability (which may contribute to the findings that men perpetrate violence less frequently than women) (Shorey et al. 2008). The systematic review by Jennings et al. (2017) found similar dating violence perpetration rates by sexes: 9 to 37% in girls and 6 to 21% in boys. In the case of online dating violence, several studies do not find significant differences between boys and girls (Bennet et al. 2011; Borrajo et al. 2015; Didden et al. 2009; Romo-Tobón et al. 2020), indicating that both perpetrate and suffer this kind of dating violence to the same extent. Other studies show higher online dating violence victimization in girls (Yahner et al. 2015; Zweig et al. 2013, 2014), while further research indicates higher victimization in boys (Cutbush et al. 2018; Hinduja & Patchin, 2021).

Considering that almost 90% of people start their intimate relationships between the ages of 16 and 20, it is vital to know the prevalence of perpetration and victimization according to age (Muñoz-Rivas et al. 2007). Regarding offline dating violence, recent studies indicated that 59.2% of young people with an average age of 19 had experienced some form of violence (Hébert et al. 2019). Some studies suggest that older adolescents are at higher risk for dating violence (Dosil-Santamaria et al. 2022), while others point to a higher risk of victimization at younger ages, particularly in girls (Bonomi et al. 2012), and less violent behavior (Pacheco et al. 2017; Smith et al. 2003). However, it is important to focus on the age rate of the studies. In this sense, Foshee et al. (2009) found that the trajectory of dating violence in young couples over time was not linear but somewhat curved and that it tends to decrease from the age of 16–17.

Regarding online dating violence and age, existing research does not provide clear answers, studies found no differences in online dating violence by age (Smith et al. 2008), and other studies showed higher rates of online dating violence in older adolescents (Sánchez et al. 2015).

### Association between online and offline dating violence

According to Riggs and O’Leary’s (1989) contextual model of dating abuse, those who experience aggression in one context (i.e., offline dating violence) would be more likely to perpetrate aggression in another context (e.g., online dating violence). Online and offline dating violence are interrelated, as they share some characteristics, especially those related to psychological violence, such as control, humiliation, jealousy, isolating the other person from his/her close environment, and threats (Caridade et al. 2019; Gámez-Guadix et al. 2018; Gracia-Levia et al. 2019; Sargent et al. 2016). However, they have distinguishing characteristics. Thus, online dating violence may have higher scope (Van Ouytsel et al. 2016) (for example, when disseminating compromising photos as a form of revenge), consequently, a higher risk of repeated victimization through the social media (Stonard, 2020) (for example, taking into account the difficulty of making photos disappear from the Internet, once they have been disseminated), exposing the victim repeatedly, even after the relationship is over (Melander, 2010).

Indeed, several studies have reported co-occurrence of both types of dating violence (Cutbush et al. 2012; Marganski & Melander, 2018), both concurrently and over time (Temple et al. 2016). Thus, nearly all respondents who experienced offline dating violence in the study by Marganski and Melander (2018), also reported having been victims of online dating violence. Still, these percentages were much lower for those who were not victims of offline dating violence but did report online dating violence. Moreover, these authors found that online violence victimization was the strongest predictor of offline violence victimization (i.e., psychological, physical, and sexual). Specifically, in the case of psychological offline violence victimization, the only significant predictor was online violence victimization. Temple et al. (2016) even suggest that online dating violence may be a vehicle to perpetrate psychological violence instead of a distinct form of abuse. Similarly, Zweig et al. (2013) asserted that social media and the Internet may be just mechanisms by which adolescents experience psychological abuse.

Studies that have explored the co-occurrence of violence have also reported co-occurrence of perpetration and victimization, known as dual violence, reciprocal abuse or bidirectionality of violence. Murford and Giordano (2008) suggest that the higher percentage of reciprocal violence in dating relationships (compared with intimate partner violence in adulthood) may be less likely to be related to the gendered nature of power dynamics; however, the adolescents’ relationships may contain other elements in their perception of intimacy that make difficult to withdraw from them (Giordano et al. 2010). The 4-year longitudinal research by Fernández-González et al. (2020) found that one key risk factor for dating violence perpetration was victimization, and for dating violence, victimization was being a perpetrator of dating violence, which highlights the relevance of reciprocal violence. Temple et al. (2016) found in their longitudinal study that online dating violence perpetration at baseline predicted online dating victimization 1 year later, which supports the notion of reciprocal online dating violence (Cutbush et al. 2010; Picard, 2007; Zweig et al. 2013), similarly to offline dating violence (Nocentini et al. 2010; O’Leary et al. 2008; Orpinas et al., 2012; Renner & Whitney, 2010). However, the mutual or reciprocal dating violence may also be the result of the presence of common characteristics that increase the odds to be a perpetrator and the victim (Reingle et al. 2014). Therefore, this only reinforces the need to deepen the knowledge of the individual characteristics of victims and perpetrators.

### Factors related to online and offline dating violence

The study of offline dating violence victims’ psychological profile has revealed that they usually show lower levels of self-esteem and that their self-concept is negatively affected (Carrascosa et al. 2016; Penado & Rodicio-García, 2017; Van Ouytsel et al. 2017). Self-esteem can even be a predictor factor of frequent victimization (Dosil-Santamaria et al. 2022). However, low self-esteem has also been linked to dating violence perpetration (Foshee et al. 2004; Lapierre et al. 2019). Regarding psychological symptomatology, internalizing symptoms such as anxiety and depression appear frequently related to intimate partner victimization (Garthe et al. 2021; Taquette & Monteiro, 2019; Zweig et al. 2013).

On the other hand, hostility is another prominent variable, given that both victims and perpetrators often show high levels of hostility in dating relationships, possibly, due to being exposed to victimization experiences during their childhood (Boivin et al. 2012; Norlander & Eckhardt, 2005). In line with the latter, theories on emotional intelligence could offer clues to understand how emotion-related facets may be linked to intimate partner violence (Fernández-González et al. 2018) and specifically to victimization, something that has mainly been investigated in peer contexts so far (Shields & Cicchetti, 2001; Spence et al. 2009). Regarding perpetrators, previous studies have linked batterers to lower scores on emotional intelligence (Shorey et al. 2015; Winters et al. 2004; Garcia-Sancho et al. 2014).

Regarding the role of online dating violence victims and aggressors, the results are not as conclusive. Among these victims, self-esteem is also severely affected. Thus, for example, some studies have found that self-esteem levels are lower among those adolescents who have suffered this specific type of violence (Jonsson et al. 2019), while it was higher among perpetrators (Smith et al. 2018). Furthermore, experiencing online dating violence has been associated with adverse psychological and physical outcomes, including depression and anxiety (Borrajo & Gámez-Guadix, 2016), weight change, sleep disturbances, and self-harm (Sheridan & Lyndon, 2012). Similar results were encountered by Zweig et al. (2014), who found higher levels of depressive symptomatology and hostility among victims and aggressors. Regarding emotional regulation, findings from general studies on suffered violence (Shields & Cicchetti, 2001; Spence et al. 2009) point to possible worse regulation strategies in victims of online intimate partner violence, although there are no precise results at the moment.

Concerning the role of online perpetration, this has been positively correlated with hostility (Deans & Bhogal, 2019; Redondo et al. 2019) and anxiety (Villora et al. 2021). However, some studies have subtle negative effects, and the perpetration of online dating abuse can be moderated by emotional regulation (Shorey et al. 2015).

### The present study

As can be seen from the previous paragraphs, there are significant gaps in the understanding of the characteristics associated with online and offline dating violence with respect to some important variables (gender, age, co-occurrence of these types of violence, relationships between perpetration and victimization in each of the forms of violence), as well as a lack of conclusive studies on the roles of online and offline perpetrators and victims. On the other hand, it could be of interest to include in the study other variables such as the educational level or nationality of the parents, which have not been studied as much and could be significant for the understanding of the phenomenon.

This study has three main aims: (1) to explore and compare the prevalence of online and offline dating violence regarding the sex and age of the participants, (2) to investigate the co-occurrence of online and offline dating violence which will be analyzed to improve the understanding of these two realities, and (3) to analyze differences on the roles of online and offline victims and perpetrators in several relevant psychosocial variables (self-esteem, hostility, emotional intelligence, subjective distress, and general functioning). Related to these aims, the following hypotheses are put forward: (1) There will be a high prevalence of victims and perpetrators for this age group. (2) There will not be significant differences based on gender or age for victimization or perpetration. (3) There will be reciprocal associations between online and offline cyberdating victimization and perpetration. (4) While there will be variables associated to all roles (such as self-esteem), there will be different variables associated with each of the analyzed roles (offline victims, offline perpetrators, online victims, and online perpetrators).

## Method

### Participants

A total of 341 young university students from the Autonomous Community of the Basque Country, Spain, participated in this study. Participants ranged in age from 17 to 24 years (*M* = 19.36, *SD* = 3.55); 75.3% (*n* = 257) were girls and 24.7% (*n* = 84) were boys. Most were from the Basque Country; 98.8% (*n* = 337) and 1.2% (*n* = 4) were of foreign origin. Regarding family composition, 78.9% (*n* = 266) had married parents, 13.6% (*n* = 46) separated, 4.7% (*n* = 16) widowed father or widowed mother, 2.1% (*n* = 7) other family structures, and 0.6% (*n* = 2) single-parent family.

### Measures

*Adolescent Social Media Partner Violence Scale* (E-VPA) (Cava & Buelga, 2018): This scale obtains measures of aggression and control suffered and perpetrated by the couple through social media and mobile devices. The scale comprises 20 items, with 10 measuring experiences of online dating victimization (five of direct violence, like “My girl/boyfriend has insulted or threatened me in private” and five of control, like “My girl/boyfriend monitors whether I’m online on my phone or connected up to the social media”) and the other 10 measuring online dating violence perpetrated against one’s partner (five of direct violence, “I have insulted or threatened my girl/boyfriend in private,” and five of control, “I monitor whether my girl/boyfriend is online on their phone or connected up to the social media”). Items are rated on a Likert-type scale from 1 (never) to 4 (always). Thus, the higher the score, the higher the online dating violence or victimization. The scale had high levels of overall internal consistency (*α* = 0.82): *α* = 0.67 for the online dating violence perpetration subscale and *α* = 0.80 for the online dating violence victimization scale.

*Conflict in Adolescent Dating Relationships Inventory* (CADRI; Wolfe et al. 2001, Spanish adaptation of Fernández-Fuertes et al. 2006): The questionnaire consists of 17 items that analyze the different types of victimization of violence and 17 items that relate to perpetration: relational (“I said things to his/her friends about him/her to make them go against him/her”), verbal–emotional (“I brought up in conversation something bad that he/she had done in the past”), and physical violence/victimization (“I pushed him/her or I shook him/her”). Participants are asked to identify how often they have experienced these situations in their dating relationships: never (this has not happened in our relationship), rarely (1 or 2 times), sometimes (between 3 and 5 times), or frequently (6 or more times). In the present study, the reliability of the victimization subscale was 0.83, and for the perpetration subscale, it was 0.78. The total alpha coefficient of this sample was 0.89.

*Rosenberg self-esteem scale* (EAR; Rosenberg, 1965; Spanish validation by Atienza et al. 2000): This scale measures self-esteem by means of 10 items, of which five are written in negative (e.g., “I feel I do not have much to be proud of”) and the rest in positive (e.g., “On the whole, I am satisfied with myself.”). Items are scored on a Likert-type scale from 1 (strongly agree) to 4 (strongly disagree). This scale has an adequate internal consistency with a Cronbach’s alpha of 0.77.

*Hostility subscale of the SCL-90-R* (Derogatis & Cleary, 1977; Spanish adaptation by González de Rivera et al. 1989): The Hostility dimension refers to thoughts, feelings, and actions characteristic of the presence of negative effects of anger (5 items). Participants must answer each item according to a scale where (0 = not at all, and 4 = very or extremely), and according to the discomfort they have experienced during the previous week, including the day on which the questionnaire is completed. The through-going question is: “How much has the actual problem distressed or bothered you?”, and an example of item “having urges to beat, injure, or harm someone.” The total alpha coefficient of this sample was 0.76.

*Clinical Outcomes in Routine Evaluation-Outcome Measure* (CORE-OM; Evans et al. 2000, Spanish version by Feixas et al. 2012): It is a self-report questionnaire composed of 34 items with a 5-category multiple-choice format (0 = never; 1 = only sometimes; 2 = sometimes; 3 = often; 4 = almost always or always) that assesses the general psychological state of the participant based on four dimensions: subjective distress (the opposite of well-being) (“I have felt like crying”), problems/symptoms (“I have felt tense, anxious or nervous”), general functioning problems (“I have felt humiliated or shamed by other people”), and risk (“I have thought of hurting myself”) (Lyne et al. 2006). For the correction of the scale, some of the items have to be reversed so that the higher the score in each subscale means more distress (i.e., less well-being), more problems and symptoms, more general functioning problems, and greater risk behaviors (e.g., non-suicidal self-injury). Cronbach’s alpha ranged between 0.69 and 0.81 depending on the scale and 0.91 for the scale as a whole.

*Trait Meta-Mood Scal*e (TMMS; Salovey et al. 1995; Spanish adaptation by Fernández-Berrocal et al. 2004): This 24 item-instrument assesses intrapersonal aspects of Emotional Intelligence, consisting of three dimensions: (1) emotional attention (level of belief about emotional focus) (e.g., “I pay a lot of attention to how I feel”); (2) emotional clarity (perception of one’s own emotions) (e.g., “I am often aware of my feelings on matter”); and (3) emotional repair (belief in being able to interrupt and regulate negative emotional states and strengthen positive ones) (e.g., “When I become upset I remind myself of all the pleasures in life”). Participants evaluate the degree to which they agree with each of the items on a 5-point Likert-type scale, with 1 = never and 5 = always. The internal consistency for each of the dimensions for this study was attention 0.90, clarity 0.92, and repair 0.87. The total alpha coefficient of this sample was 0.90.

### Procedure

The study was approved by the Ethics Committee of the UPV/EHU (M10/2018/208). To carry out this research, we contacted the teaching staff who teach on the Infant, Primary and Social Education degrees at the University of the Basque Country (Spain) and explained the aim of the study, the procedure to be followed, and the instruments to be used. Once in the classroom, the instructions for completing the questionnaires were explained aloud, and it was also made clear that participation was voluntary and anonymous and that they were free to leave the test at any time. Participants completed the questionnaires during non-teaching hours.

### Statistical analysis

First, two groups of young adults were formed according to their involvement or not in aggression and victimization behaviors in their intimate partner relationships, taking into account the cut-offs suggested by Cava and Buelga (2018) for the eVPA (i.e., being a perpetrator or victim of any conduct once or more) and Wolfe et al. (2001) for the CADRI (i.e., being a victim or perpetrator of any conduct sometimes). The frequency and percentage of students assigned to each group in each modality analyzed were calculated and their possible association with socio-demographic characteristics was investigated using the chi-squared test (*X* ^*2*^) in the case of nominal and ordinal qualitative variables and eta-squared (*ŋ* ^*2*^) for the age interval variable. Mann–Whitney *U*-tests (*Uz*) were then conducted to examine possible differences between these groups on psychosocial variables and significant results were accompanied by effect size calculations (Cohen’s *d* (*d*)) to assess their magnitude. Taking into consideration that Cohen (1988) proposed the following intervals for *d*: 0.2 to 0.4, small effect; 0.4 to 0.7, intermediate effect; and 0.8 or more, large effect. The association between the exercise/suffering of violence within each typology and between typologies was also assessed using chi-square tests. All analyses were conducted using the SPSS v.27 statistical package (IBM SPSS Statistics for Windows, Version 27.0. Armonk, NY).

## Results

### Prevalence of online and offline dating violence perpetration and victimization

Table 1 shows the prevalence of perpetration and victimization of online and offline dating violence. Approximately half of the university students indicated having exhibited some online violent behavior towards their partners (55.4%), with online control standing out, and the majority offline (80.4%), with verbal violence being the most frequent. In terms of victimization, around half reported having suffered online violence (51.6%), mostly in the form of online control, and almost three quarters of the sample suffered offline victimization (73.7%), with verbal violence predominating.

**Table 1:** Distribution of university students in terms of perpetration/victimization of different types of dating violence

	Yes *n (%)*	No *n (%)*
Online (eVPA)		
Violence perpetration (VP)	28 (8.3)	309 (91.7)
Control perpetration (CP)	180 (53.6)	156 (46.4)
Total perpetration (TP)	186 (55.4)	150 (44.6)
Violence victimization (VV)	60 (17.9)	275 (82.1)
Control victimization (CV)	165 (48.8)	173 (51.2)
Total victimization (TV)	173 (51.6)	162 (48.4)
Offline (CADRI)		
Relational violence perpetration (RVP)	16 (4.8)	318 (95.2)
Verbal violence perpetration (VVP)	266 (80.1)	66 (19.9)
Physical violence perpetration (PVP)	36 (10.8)	297 (89.2)
Total violence perpetration (TVP)	266 (80.4)	65 (19.6)
Relational violence victimization (RVV)	47 (14.2)	285 (85.8)
Verbal violence victimization (VVV)	247 (74.0)	87 (26.0)
Physical violence victimization (PVV)	21 (6.3)	312 (93.7)
Total violence victimization (TVV)	244 (73.7)	87 (26.3)

No statistically significant associations with appreciable effect sizes were found between any of the types of violence perpetration and victimization studied with gender, age, or family composition. Neither was the father’s or mother’s level of education nor nationality found to be an influence (see Table 2).

**Table 2:** Association between online and offline dating violence and gender, age and family composition

	Gender	Age	Family composition
	*|* ^*2*^ *(p)*	*ŋ* ^*2*^	*|* ^*2*^ *(p)*
Online (eVPA)			
Violence perpetration (VP)	0.29 (.592)	.049	1.03 (.905)
Control perpetration (CP)	1.66 (.197)	.036	8.03 (.190)
Total perpetration (TP)	1.34 (.247)	.027	6.62 (.157)
Violence victimization (VV)	1.17 (.279)	.029	1.25 (.868)
Control victimization (CV)	2.59 (.108)	.042	6.13 (.189)
Total victimization (TV)	1.32 (.249)	.004	7.30 (.121)
Offline (CADRI)			
Relational violence perpetration (RVP)	0.28 (.594)	.015	2.35 (.671)
Verbal violence perpetration (VVP)	4.80 (.028)	.046	3.90 (.419)
Physical violence perpetration (PVP)	5.48 (.019)	.079	3.32 (.506)
Total violence perpetration (TVP)	4.06 (.044)	.043	3.76 (.439)
Relational violence victimization (RVV)	5.66 (.017)	.021	1.95 (.744)
Verbal violence victimization (VVV)	1.24 (.265)	.090	4.10 (.393)
Physical violence victimization (PVV)	0.24 (.620)	.023	1.16 (.884)
Total violence victimization (TVV)	1.29 (.254)	.092	4.26 (.371)

### Association between online and offline dating violence perpetration and victimization

A statistically significant association was found between the perpetration or non-perpetration of online and offline violence (*χ* ^*2*^_*1*_ = 23.86, *p* < 0.001), highlighting the fact that only 14.3% of the students surveyed reported not perpetrating any type of violence on their partner, while 49.2% acknowledged having perpetrated both types of violence (see Table 3). It is worth noting that 90% of the participants who perpetrated online violence also did so offline. However, a lower percentage of students reported offline violence perpetrating online violence (61.4%).

**Table 3:** Association between types of violence and types of victimization

Perpetration					Victimization				
			Offline (CADRI)					Offline (CADRI)	
			No	Yes				No	Yes
Online (eVPA)	No	*n*	47	102	Online (eVPA)	No	*n*	71	87
		*%* within eVPA	31.5	68.5			*%* within eVPA	44.9	55.1
		*%* within CADRI	72.3	38.6			*%* within CADRI	81.6	36.1
		*%* of total	14.3	31.0			*%* of total	21.6	26.5
		Standardized residual	3.2	− 1.6			Standardized residual	4.5	− 2.7
	Yes	*n*	18	162		Yes	*n*	16	154
		*%* within eVPA	10.0	90.0			*%* within eVPA	9.4	90.6
		*%* within CADRI	27.7	61.4			*%* within CADRI	18.4	63.9
		*%* of total	5.5	49.2			*%* of total	4.9	47.0
		Standardized residual	− 2.9	1.5			Standardized residual	− 4.3	2.6

A similar pattern was found in the case of victimization (*χ* ^*2*^_*1*_ = 53.02, *p* < 0.001; see Table 2). 21.6% reported never having been victims of intimate partner violence, and 47.0% reported having suffered online and offline violence. Of the participants who suffered violence online, 90.6% also suffered violence offline, but only 63.9% of those who suffered violence offline also suffered violence online.

An association was also found between the perpetration and victimization of violence both online (*χ* ^*2*^_*1*_ = 68.23, *p* < 0.001) and offline (*χ* ^*2*^_*1*_ = 163.55, *p* < 0.001).

As shown in Table 4, 33% of the participants neither perpetrated nor received violence through the network, 15.3% only perpetrated it, 12% only received it, and 39.6% played both roles (victim-aggressor). Regarding the association between offline victimization and perpetration (see Table 4), it is noteworthy that 71.3% of university students reported having been involved in behaviors of victimization and aggression towards their intimate partners, 8.8% had exercised violence without receiving it, 2.1% had only suffered it, and 17.7% had neither exercised nor received violence.

**Table 4:** Association between online violence and victimization (eVPA) and offline violence and victimization (CADRI)

Online victimization/perpetration					Offline victimization/perpetration				
			Online violence					Offline violence	
			No	Yes				No	Yes
Online victimization	No	*n*	110	51	Offline victimization	No	*n*	58	29
		% within victimization	68.3	31.7			% within victimization	66.7	33.3
		% within violence	73.3	27.9			% within violence	89.2	11.0
		% of total	33.0	15.3			% of total	17.7	8.8
		Standardized residual	4.4	− 4.0			Standardized residual	9.8	− 4.9
	Yes	*n*	40	132		Yes	*n*	7	234
		% within victimization	23.3	76.7			% within victimization	2.9	97.1
		% within violence	26.7	72.1			% within violence	10.8	89.0
		% of total	12.0	39.6			% of total	2.1	71.3
		Standardized residual	− 4.3	3.9			Standardized residual	− 5.9	2.9

### Differences in the psychosocial profile as a function of the online and offline dating violence perpetration and victimization

Possible differences in psychosocial aspects were examined according to whether the students were involved in violence perpetration or victimization in their relationships (see Tables 5, 6, 7 and 8).

**Table 5:** Differences in psychosocial variables according to the perpetration or not of online dating violence

	Online dating violence (eVPA)														
	Violence perpetration					Control perpetration					Total				
	No		Yes			No		Yes			No		Yes		
	*M*	*SD*	*M*	*SD*	*Uz (p)*	*M*	*SD*	*M*	*SD*	*Uz (p)*	*M*	*SD*	*M*	*SD*	*Uz (p)*
Self-esteem	30.09	5.45	27.93	5.86	− 1.94 (.052)	31.06	5.36	28.95	5.47	− 3.52 (< .001)*	31.40	5.33	28.89	5.51	− 3.88 (< .001)*
Hostility	10.74	3.90	12.04	4.33	1.66 (.096)	9.82	3.47	11.73	4.13	4.72 (< .001)*	9.67	3.40	11.63	4.11	5.04 (< .001)*
Attention	29.01	6.54	29.29	6.97	0.41 (.678)	28.61	6.34	29.34	6.74	1.14 (.254)	28.74	6.37	29.06	6.75	1.08 (.280)
Clarity	25.41	6.96	24.48	8.05	− 0.64 (.519)	26.01	6.88	24.76	7.18	− 1.62 (.105)	26.11	6.53	24.63	7.22	− 2.21 (.027)*
Repair	27.06	6.30	25.57	6.91	− 1.00 (.316)	27.97	6.25	26.08	6.33	− 2.63 (< .001)*	28.25	6.11	26.20	6.26	− 2.76 (.006)*
Subjective distress	12.76	2.21	13.32	2.34	1.23 (.218)	12.71	2.27	12.90	2.20	0.98 (.328)	12.56	2.25	12.89	2.15	1.19 (.232)
Problems/symptoms	28.41	7.62	31.79	7.39	2.33 (.019)*	26.68	7.39	30.42	7.48	4.91 (< .001)*	26.23	7.08	30.54	7.57	5.19 (< .001)*
General functioning	33.04	3.82	34.07	3.71	1.35 (.175)	32.79	3.67	33.42	3.93	1.59 (.112)	32.88	3.59	33.41	3.87	1.17 (.242)
Risk	4.44	1.46	4.39	1.10	0.18 (.854)	4.30	1.16	4.56	1.63	1.51 (.131)	4.24	0.86	4.56	1.63	1.55 (.121)

Note: * = *p* < .05, ** = *p* < 0.01, *** = *p* < .001

**Table 6:** Differences in psychosocial variables according to the perpetration or non-perpetration of offline dating violence

	Offline dating violence (CADRI)																			
	Relational violence					Verbal violence					Physical violence					Total				
	No		Yes			No		Yes			No		Yes			No		Yes		
	*M*	*SD*	*M*	*SD*	*Uz (p)*	*M*	*SD*	*M*	*SD*	*Uz (p)*	*M*	*SD*	*M*	*SD*	*Uz (p)*	*M*	*SD*	*M*	*SD*	*Uz (p)*
Self-esteem	30.00	5.55	28.47	5.51	− 1.06 (.286)	31.29	5.06	29.58	5.62	− 2.10 (.035)*	30.01	5.49	29.50	6.00	− 0.33 (.744)	31.24	5.10	29.65	5.64	− 1.91 (.056)
Hostility	10.64	3.87	14.38	4.16	− 3.52 (< .001)*	9.27	3.03	11.21	4.07	− 3.73 (< .001)*	10.63	3.82	12.28	4.67	− 2.08 (.037)*	9.37	3.04	11.15	4.06	− 3.49 (< .001)*
Attention	28.89	6.53	31.25	7.22	− 1.74 (.080)	28.67	5.99	29.07	6.71	− 0.70 (.480)	29.02	6.55	28.80	6.89	− 0.01 (.995)	28.67	6.06	28.99	6.72	− 0.53 (.592)
Clarity	25.23	7.02	27.19	7.82	0.98 (.324)	25.56	6.47	25.30	7.19	− 0.40 (.686)	25.28	6.96	25.63	7.99	− 0.24 (.810)	25.40	6.38	25.24	7.11	− 0.22 (.820)
Repair	26.94	6.36	25.93	6.71	− 0.43 (.663)	28.00	6.85	26.64	6.22	− 1.47 (.141)	26.84	6.36	27.23	6.57	− 0.43 (.663)	27.90	6.96	26.88	6.07	− 1.38 (.168)
Subjective distress	12.81	2.19	13.25	2.49	− 0.26 (.795)	12.32	2.36	12.94	2.15	− 2.21 (.027)*	12.76	2.19	13.31	2.27	− 1.23 (.217)	12.30	2.37	12.86	2.14	− 2.36 (.018)*
Problems/symptoms	28.62	7.58	30.75	8.54	− 0.96 (.337)	25.44	6.27	29.52	7.75	− 4.00 (< .001)*	28.48	7.59	30.47	7.84	− 1.72 (.086)	25.52	6.41	29.49	7.75	− 3.90 (< .001)*
General functioning	33.13	3.68	33.50	5.43	− 0.46 (.640)	32.05	3.49	33.44	3.80	2.91 (.004)*	33.00	3.74	34.28	3.85	− 2.14 (.032)*	31.98	3.49	33.49	3.76	− 3.04 (.002)*
Risk	4.46	1.47	4.13	0.50	− 0.86 (.388)	4.24	0.95	4.50	1.54	− 1.16 (.246)	4.46	1.49	4.33	1.01	− 0.37 (.709)	4.25	0.97	4.46	1.44	− 1.12 (.261)

Note: * = *p* < .05, ** = *p* < 0.01, *** = *p* < .001

**Table 7:** Differences in psychosocial variables according to whether or not the victim suffered from online dating violence

	Online victimization (eVPA)														
	Violence victimization					Control victimization					Total				
	No		Yes			No		Yes			No		Yes		
	*M*	*SD*	*M*	*SD*	*Uz (p)*	*M*	*SD*	*M*	*SD*	*Uz (p)*	*M*	*SD*	*M*	*SD*	*Uz (p)*
Self-esteem	30.19	5.44	28.88	5.87	− 1.49 (.134)	30.21	5.55	29.68	5.53	− 0.51 (.609)	30.28	5.58	29.63	5.59	− 0.73 (.466)
Hostility	10.48	3.76	12.14	4.41	− 2.67 (.007)*	10.01	3.42	11.58	4.26	− 3.73 (< .001)*	9.99	3.49	11.51	4.19	− 3.65 (< .001)*
Attention	28.67	6.40	30.16	7.21	− 1.64 (.100)	29.04	6.67	28.84	6.49	− 0.07 (.941)	28.97	6.58	28.93	6.63	− 0.13 (.893)
Clarity	25.32	6.79	24.75	7.76	− 0.99 (.319)	25.31	6.94	25.12	7.02	− 0.47 (.640)	25.38	6.71	25.03	7.22	− 0.96 (.335)
Repair	27.22	6.30	26.26	6.07	− 1.26 (.207)	27.81	6.11	26.26	6.34	− 1.32 (.187)	27.90	6.23	26.16	6.20	1.97 (.049)*
Subjective distress	12.62	2.27	13.19	1.95	− 2.11 (.034)*	12.52	2.48	12.94	1.91	− 1.86 (.062)	12.52	2.43	12.96	1.96	− 2.03 (.042)*
Problems/symptoms	27.74	7.42	32.26	7.68	− 3.94 (< .001)*	27.10	7.41	30.06	7.64	− 4.01 (< .001)*	27.01	7.35	30.08	7.66	− 4.28 (< .001)*
General functioning	33.01	3.59	33.68	4.67	− 0.88 (.378)	33.10	3.47	33.18	4.14	− 0.83 (.408)	33.10	3.42	33.27	3.92	− 0.89 (.373)
Risk	4.37	1.28	4.61	1.60	− 1.29 (.196)	4.39	1.42	4.44	1.27	− 1.33 (.184)	4.42	1.48	4.43	1.25	− 0.93 (.353)

Note: * = *p* < .05, ** = *p* < 0.01, *** = *p* < .001

**Table 8:** Differences in psychosocial variables as a function of suffering or not from offline dating violence

	Offline dating victimization (CADRI)																			
	Relational violence victimization					Verbal violence victimization					Physical violence victimization					Total				
	No		Yes			No		Yes			No		Yes			No		Yes		
	*M*	*SD*	*M*	*SD*	*Uz (p)*	*M*	*SD*	*M*	*SD*	*Uz (p)*	*M*	*SD*	*M*	*SD*	*Uz (p)*	*M*	*SD*	*M*	*SD*	*Uz (p)*
Self-esteem	30.00	5.55	28.47	5.51	− 0.18 (.859)	31.29	5.06	29.58	5.62	− 2.23 (.026)*	30.01	5.49	29.50	6.00	− 0.72 (.471)	31.22	5.46	29.47	5.57	− 2.24 (.025)*
Hostility	10.64	3.87	14.38	4.16	− 2.24 (.025)*	9.27	3.03	11.21	4.07	− 3.63 (< .001)*	10.63	3.82	12.28	4.67	− 1.05 (.292)	9.58	3.26	11.23	4.08	− 3.70 (< .001)*
Attention	28.89	6.53	31.25	7.22	− 0.01 (.992)	28.67	5.99	29.07	6.71	− 0.34 (.736)	29.02	6.55	28.80	6.89	− 0.46 (.648)	28.93	5.77	28.96	6.89	− 0.40 (.690)
Clarity	25.23	7.02	27.19	7.82	− 0.91 (.363)	25.56	6.47	25.30	7.19	− 0.66 (.506)	25.28	6.96	25.63	7.99	− 0.25 (.801)	25.72	6.21	25.00	7.23	− 0.71 (.479)
Repair	26.94	6.36	25.93	6.71	− 0.26 (.793)	28.00	6.85	26.64	6.22	− 1.74 (.082)	26.84	6.36	27.23	6.57	− 1.55 (.121)	28.17	6.32	26.56	6.20	− 1.77 (.076)
Subjective distress	12.81	2.19	13.25	2.49	− 1.36 (.173)	12.32	2.36	12.94	2.15	− 2.31 (.021)*	12.76	2.19	13.31	2.27	− 1.16 (.248)	12.36	2.34	12.89	2.14	− 2.34 (.019)*
Problems/symptoms	28.62	7.58	30.75	8.54	− 1.85 (.065)	25.44	6.27	29.52	7.75	− 4.93 (< .001)*	28.48	7.59	30.47	7.84	− 2.26 (.024)*	25.42	6.90	29.79	7.60	− 4.91 (< .001)*
General functioning	33.13	3.68	33.50	5.43	− 2.23 (.026)*	32.05	3.49	33.44	3.80	− 2.26 (.024)*	33.00	3.74	34.28	3.85	− 1.16 (.246)	32.60	3.57	33.40	3.71	− 2.27 (.023)*
Risk	4.46	1.47	4.13	0.50	− 1.64 (.101)	4.24	0.95	4.50	1.54	− 0.61 (.542)	4.46	1.49	4.33	1.01	0.88 (.380)	4.27	0.94	4.48	1.49	− 0.65 (.518)

Note: * = *p* < .05, ** = *p* < 0.01, *** = *p* < .001

The results showed that people who exercised online violence towards their partners presented more psychological problems/symptoms (*d* = 0.26). Those who exercised online control had lower self-esteem (*d* = 0.39) and reparation (*d* = 0.29) and higher scores in hostility (*d* = 0.53) and psychological problems/symptoms (*d* = 0.56). Without distinguishing by subtypes, it was observed that those who perpetrated some type of violence had significantly lower scores in self-esteem (*d* = 0.43), clarity or understanding of their own emotional state (*d* = 0.25), and emotional repair or regulation (*d* = 0.31) but, above all, higher scores in hostility (*d* = 0.57) and psychological problems/symptoms (*d* = 0.59).

Offline dating violence perpetration was related to higher scores in hostility (*d* = 0.39), general subjective distress (*d* = 0.26), psychological problems/symptoms (*d* = 0.44), and difficulties in general functioning (*d* = 0.34). However, if the violence was relational, the differences were only statistically significant in hostility (*d* = 0.39), if it was physical violence in hostility (*d* = 0.23) and general functioning (*d* = 0.24) and if it was verbal violence, the differences were lower in self-esteem (*d* = 0.23) and higher in hostility (*d* = 0.42), subjective distress (*d* = 0.24), problems/symptoms (*d* = 0.45), and general functioning (*d* = 0.32).

Being a victim of online violence was related to having significantly higher scores in hostility (*d* = 0.41), subjective distress (*d* = 0.22), emotional problems (*d* = 0.48), and lower emotional regulation capacity (*d* = 0.22) than those students who had not experienced it so far. By subtypes, general victimization was associated with greater hostility (*d* = 0.30), subjective distress (*d* = 0.23), and emotional problems (*d* = 0.44). Receiving online control translated into significantly higher scores on hostility (*d* = 0.41) and emotional problems (*d* = 0.45).

Offline victimization was characterized by lower self-esteem (*d* = 0.25) and higher hostility (*d* = 0.42), subjective distress (*d* = 0.26), emotional problems/symptoms (*d* = 0.56), and more difficulties in general functioning (*d* = 0.25). Relational victimization resulted in differences in hostility (*d* = 0.25) and general functioning (*d* = 0.25). Verbal victimization had lower self-esteem (*d* = 0.25) and higher hostility (*d* = 0.41), subjective distress (*d* = 0.26), emotional problems/symptoms (*d* = 0.56) and more difficulties in general functioning (*d* = 0.25), and physical victimization had higher psychological problems/symptoms (*d* = 0.25).

## Discussion

One of the purposes of this study was to analyze the prevalence of violence in the romantic relationships of young university students both online and offline and to see its association with gender, age, and parents’ level of education and nationality. The prevalence rates of online dating violence in this study are higher than those found in a national study also with university students (Borrajo et al. 2015). In contrast, Borrajo et al. (2015) reported that 82% of their participants exercised online control, a higher percentage than that found in our study. In terms of experiencing online dating violence or online control, the study by Borrajo et al. (2015) also showed a higher percentage in some cases (75% of online dating violence victimization) than that found in our study (51.6%). In relation to offline dating violence, our study reports percentages of 80.4% for perpetration and 73.7% for victimization, higher than those found in other studies with young participants up to 22 years of age (Martínez et al. 2016). The verbal-emotional violence has been the most prevalent, with 80.1% for perpetration and 74% for victimization, which indicates its widespread use among young people. These percentages are similar to other studies conducted in Spain (Cava et al. 2015) and even quite lower than others that revealed rates of 90% (Muñiz-Rivas et al. 2007). Regarding physical offline dating violence, the prevalence rates in this study are like those found in previous studies (Foshee et al., 2007; Niolon et al. 2015; Wincentak et al. 2017).

As for gender, no statistically significant associations were found in accordance with previous studies (Borrajo et al., 2015; Didden et al. 2009; Romo-Tobón et al. 2020). Both partners often indicate in these self-reports that they perpetrate and suffer violence offline and online. It would be necessary to further analyze whether the perpetrated violence is due to perceived emotional abuse, to the scarce recognition of dating violence forms in romantic relationships or even to the naturalization and normalization of violent behaviors as part of the imaginary of romantic relationships in adolescents and young people.

Age has not shown statistically significant associations in this study either. However, several studies point out that the trajectory of violence in dating relationships is not linear but curved and that although violence may decrease as they get older (Foshee et al. 2009), violence is much more severe at older ages (González-Ortega et al., 2008). Therefore, future studies should explore samples with a larger age rate.

The results of the present study have confirmed that offline and online dating violence are closely related. Almost half of the participants acknowledged experiencing both online and offline violence (49.2%) or having been a victim of both types of violence (47%), a percentage slightly lower than the victimization data found by Gracia-Leiva et al. (2020) (56.8%).

Regarding the overlap between online and offline violence, most participants (90%) who reported having experienced or been victims of online violence also reported having been victims of offline violence. Still, this percentage was 30% lower in the case of those who experienced or had been victimized offline and who reported having been victimized online as well. As in the Marganski and Melander (2018) study, the percentage of participants who are not victims of offline violence but report having been victims of online violence is very low (close to 9%). The conclusion to be drawn from these results might be that online dating violence (perpetrated and suffered) also entails offline violence, but not all offline violence is associated with online violence. These results further confirm the idea that online and offline violence are two different entities and should be treated as such. Future longitudinal studies should further explore the longitudinal nature of online dating violence, which would be of great interest to the design of dating violence prevention programs.

Regarding reciprocal or dual violence, the results of the present study suggest that this reciprocity is greater for offline violence (71.3%) than for online violence (39.6%), although it occurs in both types of violence, prevalence rates similar to other studies (Gray & Foshee, 1997; Whitaker et al. 2007; Zweig et al. 2013). These results indicate that violence is frequently a way of solving problems in dating relationships, where both are perpetrators and victims. This could lead to an escalation of violence, where abuse may escalate from verbal to physical, which plays an important role in dating violence (Wekerle & Wolf, 1999). The reciprocal violence rates differ considerably from offline to online dating violence, which once again confirms the idea that offline and online violence are different or that they respond to different patterns. With the data collected in the present study, it is not possible to go any further in understanding why these differences in the reciprocity of offline and online violence occur; nevertheless, future studies should also examine whether such violence is offensive or defensive violence, as well as the frequency and severity of violence being perpetrated.

The results indicate that although both types of violence (offline and online) are closely related, they are distinct realities that should be studied separately. Future research could further examine the mechanisms that underlie these online and offline dating violence and how they differ from each other (Schokkenbroek et al. 2022).

The third objective of the study was to investigate in greater depth the online and offline victim and perpetrators’ roles separately in relation to some personal and symptomatological relevant variables under the hypothesis that given the specificity of these two types of violence (both when perpetrated and suffered), differences would be found in them. Considering the two victim roles (online-offline) analyzed in the study, results show that they are quite similar in almost all the tested variables. Thus, for example, in both cases, those who suffered violence show higher levels of hostility, and this is true regardless of the type of violence to which the victim is exposed (direct, control, physical, psychological).

Hostility has already been associated with dating violence victimization in adolescents (Tourigny et al. 2006), and according to some authors, can be understood as a protective or a reactive measure following aggression initiated by the partner (Boivin et al. 2012). Hence, hostile victimized girls could more easily accept violence as a self-defense measure and perceive violence as an instrument to gain their way in an argument with a partner (Leisring, 2009).

On the other hand, both online and offline dating victims showed higher levels of psychological distress (CORE-OM), i.e., they reported greater subjective distress, greater emotional problems (e.g., anxiety, depression, trauma, and physical symptoms), and greater indicators of “low risk” (thinking about harming themselves, or that it would be better to be dead). The presence of internalizing symptoms such as anxiety and depression are frequent among victims of offline dating violence (Garthe et al. 2021; Taquette & Monteiro, 2019), as well as among those who suffer online dating violence (Borrajo & Gámez-Guadix, 2016). Some studies have even found higher symptomatology levels in the latter, perhaps due to the lack of geographical and temporal limits that characterize these forms of communication, which would contribute to aggravate the consequences (Borrajo & Gámez-Guadix, 2016). Self-harm symptoms have been also reported in other studies (Sheridan & Lyndon, 2012), although in this one they are limited to the presence of cognitions or ideas such as those previously indicated. It should be noted that in the case of offline victims, they also reported more difficulties in general functioning (intimate relationships, social relationships, and other aspects of daily functioning). Future studies should clarify whether, as might intuitively be expected, offline violence intrudes more on relationships with family, partners, and peers.

Finally, there are two variables in which differences are observed in the online and offline roles of victims: self-esteem and emotional regulation. In this study, self-esteem appeared as a significant variable among offline victims, but not among online victims. In general terms, self-esteem is negatively affected in victims of violence of both types (Jonsson et al. 2019; Penado & Rodicio-García, 2017; Van Ouytsel et al. 2017), and this may be due to two reasons: those who do not value themselves may more easily tolerate aggressions from their partners (González et al. 2008), or being exposed to this type of violent relationships ends up undermining the victim’s self-esteem. The present study suggests that low self-esteem is a significant variable in the characteristics of offline violence victims, perhaps because offline attacks are harsher or have a greater impact on self-esteem, something that should be further investigated. Likewise, low emotional regulation was a significant variable only in the online dating victim role; specifically, they scored lower on the belief about being able to interrupt and regulate negative emotional states and strengthen positive ones. Emotional regulation difficulties had already been associated with victimization in intimate partner violence (Capaldi et al. 2012; Lehrer et al. 2006). Perhaps this capacity is more impaired among online victims, given that online violence, occurring at any time and place, may leave them unable to cut it off or stop it, resulting in continuous emotional dysregulation.

Regarding perpetrators, both online and offline aggressors presented higher scores on hostility and on the problems and symptoms scales than non-aggressors. However, offline aggressors (but not online perpetrators) had also higher scores on distress and lower general functioning. This is consistent with several studies that pointed out that both offline and online offenders have higher levels of hostility (Boivin et al. 2012; Deans & Bhogal, 2019; Norlander & Eckhardt, 2005; Redondo et al. 2019; Zweig et al. 2014) and more psychological problems, such as anxiety or depression (Shorey et al. 2015; Villora et al. 2021; Zweig et al. 2014).

Additionally, online (but not offline) perpetrators showed lower self-esteem scores. This is partly consistent with several studies that found lower self-esteem in offline perpetrators (Foshee et al. 2004; Lapierre et al. 2019); however, this study only found significant differences between online perpetrators and non-perpetrators which contrast with the study of Smith et al. (2018). This may be because, as pointed out by Patchin and Hinduja (2010), the relationship between aggressors and self-esteem is more variable than in victims and would grant further study.

Lastly, this study found that online aggressors have significantly lower scores in emotional intelligence (clarity and repair) than non-online aggressors, which is consistent with the literature that suggests that offenders have lower emotional intelligence (Shorey et al. 2015; Winters et al. 2004). It may be that by presenting a lower capacity for clarity and repair, aggressors should be targets of future intervention programs as understanding their own emotions (clarity) and regulating their own emotions (repair) can be factors of higher relevance to prevent and stop the perpetration of online dating abuse.

To conclude, some limitations of the study should be pointed out: first, the intentional selection of the sample, university students from the Basque Country (Spain). Future studies should recruit larger samples from different cultures or countries, in order to obtain more generalizable results. Secondly, the measurement, as it is based exclusively on self-reporting. It would be interesting to collect information from both members of the couple, in order to contrast the results. In addition, the results could be partly biased by social desirability. Given that there is increasing social awareness and concern about the issue of violence in intimate partner relationships, it could be that the participants tried to mask their true behaviors, in order to provide a more positive self-image. Lastly, these results come from a cross-sectional study and therefore no causality can be implied from our results, making it possible that the characteristics of each role precede their role in the dating or cyber dating abuse role.

On the other hand, future studies could carry out a selection of variables that contemplate more relevant variables of the personality of victims and aggressors, since these characteristics are quite stable and not so dependent on the context or experiences, and could therefore offer a better delimitation of the characteristics of victims and aggressors. Moreover, this manuscript has not addressed the double role of victim-perpetrators that could be a distinct role that should be addressed in future studies.

Lastly, complementing the information collected with qualitative methodologies, such as interviews or focus groups, would give us more detailed information on the relationship dynamics between young people.

Despite these limitations, the study has interesting implications that should be taken into account. Thus, these results could be taken into account in order to raise awareness among university students about online and offline dating violence and also to raise awareness in the institutions in order to implement prevention or intervention programs, so that young people, whether victims, aggressors, or bystanders, have resources to be able to ask for help. Knowing the characteristics that differentiate aggressors and victims of dating violence (online or offline) would allow us to carry out more targeted interventions, in the sense that we know which aspects we should work on, so that victims or aggressors can change their behavior and attitudes, and thus stop the escalation of violence.

## Conclusions

In light of the results, although both phenomena co-exist and they show some overlap between each other, they show some differential characteristics for example in terms of self-esteem, emotional intelligence, and general functioning. Taking into account the differences and similarities practitioners should be aware that victims from offline cyber dating violence might be subject to online violence as well. Moreover, the wide array of variables used in this study should aid practitioners in carrying out an assessment and identifying key areas for treatment in victims of online and offline violence (such as general functioning) but also those mostly affecting one type of victim (such as self-esteem or emotional regulation).

## Data Availability

Data will me made available from the corresponding author upon reasonable request.

## Abbreviations

E-VPA: Adolescent Social Media Partner Violence Scale
CADRI: Conflict in Adolescent Dating Relationships Inventory
CORE-OM: Clinical Outcomes in Routine Evaluation-Outcome Measure
TMMS: Trait Meta-Mood Scale
VP: Violence Perpetration from the E-VPA scale
CP: Control Perpetration from the E-VPA scale
TP: Total Perpetration from the E-VPA scale
VV: Violence Victimization from the E-VPA scale
CV: Control Victimization from the E-VPA scale
TV: Total Victimization from the E-VPA scale
RVP: Relational Violence Perpetration from CADRI scale
VVP: Verbal-Emotional Violence Perpetration from CADRI scale
PVP: Physical Violence Perpetration from CADRI scale
TVP: Total Violence Perpetration from CADRI scale
RVV: Relational Violence Victimization from CADRI scale
VVV: Verbal-Emotional Violence Victimization from CADRI scale
PVV: Physical Violence Victimization from CADRI scale
TVV: Total Violence Victimization from CADRI scale
